# The Design, Modeling, and Experiment of a Novel Diving-Beetle-Inspired Paddling Propulsion Robot

**DOI:** 10.3390/biomimetics10030182

**Published:** 2025-03-14

**Authors:** Jiang Ding, Jingyu Li, Tianbo Lan, Kai He, Qiyang Zuo

**Affiliations:** 1 School of Mechanical Engineering, Guangxi University, Nanning 530004, China; jding@gxu.edu.cn (J.D.); jy.li10@siat.ac.cn (J.L.); tb.lan@siat.ac.cn (T.L.); 2 Shenzhen Institutes of Advanced Technology, Chinese Academy of Sciences, Shenzhen 518055, China; kai.he@siat.ac.cn; 3 Shenzhen Key Laboratory of Precision Engineering, Shenzhen 518055, China

**Keywords:** bionic robot, diving beetle, paddle propulsion, underwater robot, propulsion performance

## Abstract

Bionic paddling robots, as a novel type of underwater robot, demonstrate significant potential in the fields of underwater exploration and development. However, current research on bionic paddling robots primarily focuses on the motion mechanisms of large organisms such as frogs, while the exploration of small and highly agile bionic propulsion robots remains relatively limited. Additionally, existing biomimetic designs often face challenges such as structural complexity and cumbersome control systems, which hinder their practical applications. To address these challenges, this study proposes a novel diving-beetle-inspired paddling robot, drawing inspiration from the low-resistance physiological structure and efficient paddling locomotion of diving beetles. Specifically, a passive bionic swimming foot and a periodic paddling propulsion mechanism were designed based on the leg movement patterns of diving beetles, achieving highly efficient propulsion performance. In the design process, a combination of incomplete gears and torsion springs was employed, significantly reducing the driving frequency of servos and simplifying control complexity. Through dynamic simulations and experimental validation, the robot demonstrated a maximum forward speed of 0.82 BL/s and a turning speed of 18°/s. The results indicate that this design not only significantly improves propulsion efficiency and swimming agility but also provides new design insights and technical references for the development of small bionic underwater robots.

## 1. Introduction

Bionic underwater paddling robots represent one of the key technological approaches in the field of marine exploration [1,2,3]. By mimicking the efficient locomotion mechanisms of aquatic organisms, these robots demonstrate significant application potential and value in areas such as the exploration of marine resources [4,5,6]. The design of bionic paddling robots draws inspiration from the motion mechanisms of aquatic creatures, aiming to replicate the highly efficient propulsion strategies observed in nature. Among various underwater organisms, swimming animals driven by limbs exhibit exceptional propulsion efficiency due to their unique limb structures and movement patterns [7,8,9]. For example, the hind limbs of many aquatic species of paddling possess robust muscle tissues and high explosive power, enabling rapid and forceful movement. During swimming, the webbed toes of these organisms expand to increase the surface area, thus significantly enhancing thrust generation and facilitating efficient paddling [10,11]. The design of bionic paddling propulsion robots is based on this biological inspiration, with the aim of replicating and utilizing these natural mechanisms to improve underwater mobility and performance.

Researchers have conducted extensive studies on large paddling organisms. For example, the National University of Singapore designed a bionic robot inspired by frogs. The robot foot actuator can increase its projected area by 66% under high pressure, significantly improving the average maximum thrust by 34.5%, and the average robot swimming speed can reach 0.42 BL/s [12]. The Harbin Institute of Technology, through the study of the biological structure and limb movement characteristics of frogs, designed a bionic robot with flexible joints driven by pneumatic actuation. The robot achieves an average propulsion speed of 0.075 m/s during linear motion and an average turning speed of 15°/s [13]. Zhejiang Sci-Tech University designed a structural device inspired by the hind limbs of beavers, utilizing a combination of servo and cable-driven mechanisms to realize the paddling motion of the hind limbs of bionic beavers [14]. Most existing bionic paddling propulsion robots are primarily inspired by organisms such as frogs and beavers, while research on bionic paddling propulsion robots inspired by smaller and more agile creatures, such as diving beetles, remains relatively limited.

Compared to amphibians (such as frogs) and semiaquatic animals (such as beavers), diving beetles demonstrate superior maneuverability, capable of performing forward swimming and turning behaviors with remarkable flexibility [15,16,17]. This characteristic provides a significant reference value for the development of swimming propulsion robots. Therefore, researchers have focused on small swimming propulsion organisms, such as diving beetles, as primary research subjects. For instance, inspired by diving beetles, the Ulsan National Institute of Science and Technology in South Korea developed a small robot equipped with a pair of “swimming feet” that provide mobility. The robot optimizes the connecting rod structure to maximize the stroke angle and is driven by a single DC motor with a gear transmission system [18]. In subsequent studies, the institute designed an innovative hinged leg structure, which adaptively increases and decreases the surface area during the stroke and recovery phases, respectively, thereby achieving efficient recovery strokes and reducing resistance [19]. At Jilin University, researchers analyzed the force characteristics of diving beetles during swimming and employed computational fluid dynamics (CFD) simulation techniques to study their swimming kinematics and propulsion efficiency. Based on the hindfoot of diving beetles, they designed a biomimetic swimming propulsion mechanism. The biomimetic model was scaled up 15 times, with motors installed at each joint serving as the power source. The system consists of three motors and employs a conical gear transmission mechanism [20]. However, despite the emergence of several biomimetic diving-beetle-inspired underwater propulsion robots, these devices still face challenges such as excessive motor usage and complex control systems.

Therefore, this paper has been designed to create a biomimetic swimming propulsion robot inspired by the diving beetle. Through observations of the beetle’s body shape and swimming behavior, the robot mimics the structure and motion mechanism of the beetle’s swimming foot. Both simulations and underwater experiments were conducted to verify the robot’s effectiveness. The design employs a combination of incomplete gears and torsion springs to achieve specific motion patterns during the recovery and power strokes. This reduces the servo drive frequency, enhances the biomimetic fidelity, and improves the propulsion performance and maneuverability of the underwater robot.

The rest of this paper is organized as follows. Section 2 introduces the design inspiration and methodology of the biomimetic robot. Section 3 provides a dynamic analysis of the unilateral swimming propulsion mechanism. Section 4 presents the experimental results and discusses their implications. Finally, Section 5 summarizes this study and outlines potential directions for future research.

## 2. Design of Bionic Paddling Robots

### 2.1. Diving Beetle Motion Characterization

Through observing the morphological and movement characteristics of organisms, researchers can provide ideas and directions for biomimetic design and help summarize relevant design principles [21]. In this section, the diving beetle was taken as the research object. Its body morphology, leg structural features, and movement characteristics were observed and analyzed to provide a theoretical basis for the design of paddling propulsion mechanisms inspired by the diving beetle. Based on the body morphology and movement characteristics of the diving beetle, a paddling propulsion robot inspired by the diving beetle was designed.

**Figure 1:**
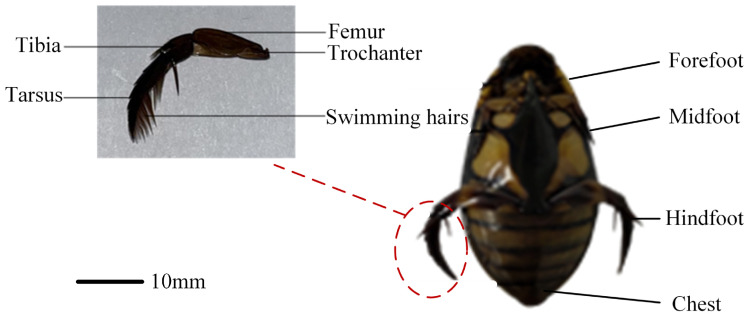
Bottom view of diving beetle.

The diving beetle exhibits a streamlined body shape, appearing nearly elliptical when viewed from the side or front. This streamlined structure reduces drag, enabling the beetle to swim quickly in water. Upon closer examination, the diving beetle’s body, when viewed from above, resembles a long ovate shape with a symmetrical appearance from left to right. Its body tapers from the head to the middle and then narrows again toward the tail. This specific morphology facilitates rapid swimming and efficient hunting in aquatic environments. The diving beetle’s paddling foot consists of several components: the trochanter, femur, tibia, tarsus, and swimming hairs located on the tarsus. As shown in Figure 1, the hindfoot’s structural features play a critical role in the beetle’s swimming ability. During swimming, the hindfoot generates propulsion through paddling motion. Notably, grooves exist between the femur, tibia, and tarsus, which function to limit the range of hindfoot movement. This constraint ensures that the hindfoot maintains an optimal paddling angle, enhancing swimming efficiency. This structural design allows the diving beetle to effectively control and adjust its hindfoot motion during swimming, thereby improving overall swimming performance.

**Figure 2:**
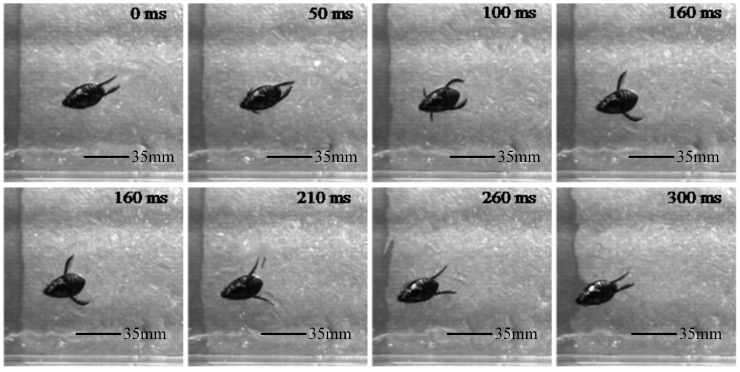
The time sequence diagram of a single cycle of forward movement of a diving beetle.

The hindfoot movement of the diving beetle exhibits a periodic variation, which can be divided into two distinct phases: the return phase and the stroke phase. During the return phase, the hindfoot swings forward towards the head, while in the stroke phase, it swings backward towards the tail. A single cycle consists of one return phase and one stroke phase. As shown in Figure 2, during the paddling propulsion process, the posture of the limb joints in the hindfoot undergoes continuous adjustments, with the joint angles dynamically changing and coordinating with each other. Within a single swimming cycle (0–300 ms), the hindfoot motion can be further divided into two primary stages: the return phase (0–160 ms, approximately 53.3% of the total cycle) and the stroke phase (160–300 ms, approximately 46.7% of the total cycle). During the return phase, the hindfoot adopts a flexed posture, thereby reducing the resistance it encounters. In contrast, during the stroke phase, the hindfoot fully extends and maintains this position to complete the stroke. Simultaneously, the swimming hairs on the hindfoot spread out, increasing the effective surface area of the stroke and consequently enhancing the propulsive force. The body length of the dragon louse is 35.74 mm and its swimming speed is 3.26 BL/s.

**Figure 3:**
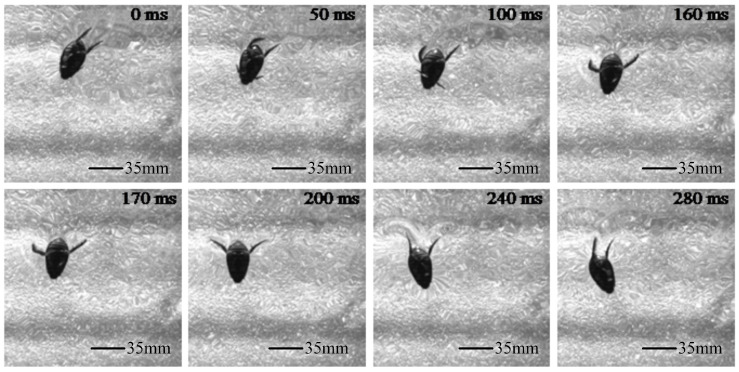
The time sequence diagram of a single cycle of turn movement of a diving beetle.

Within a single paddling cycle, the turning swimming posture sequence of the diving beetle is shown in Figure 3. As can be seen from the figure, during the paddling propulsion process, there are significant differences in the motion of the two hindfeet: one hindfoot exhibits a larger amplitude of motion and plays a primary role in turning and propulsion, while the other hindfoot has a smaller amplitude and contributes less to turning and propulsion. During the return phase (0–160 ms), the left hindfoot of the diving beetle bends with a larger bending angle, reducing resistance, while the right hindfoot first extends to paddle and then bends but with a smaller amplitude. The return phase accounts for approximately 57.1% of the entire cycle. In the stroke phase (160–280 ms), the left hindfoot fully extends and maintains this position to complete the paddling. During this phase, the stroke amplitude is large, and the swimming hairs on the hindfoot spread outward, increasing the effective paddling area and thus enhancing the propulsive force. Meanwhile, the right hindfoot first bends and then fully extends to complete the paddling, but with a smaller amplitude. The stroke phase accounts for approximately 42.9% of the entire cycle. Under the aforementioned conditions, the diving beetle rotates by an angle of 49.8° over a single cycle.

### 2.2. The Structural Design of a Bionic Paddling Robot

The diving-beetle-inspired paddling propulsion robot is based on its biological prototype, aiming to design a robot that embodies certain characteristics of the biological model. It primarily consists of a propulsion system, a control system, and an overall structure. In the overall structure, the robot’s shape and surface are designed with reference to the diving beetle’s morphology, incorporating streamlined features to reduce resistance during motion. The legs of diving beetles are paired, and symmetrical paddling enables them to adapt to harsh aquatic environments and achieve higher speeds [22]. Therefore, the design of the swimming foot structure in the diving-beetle-inspired paddling propulsion robot draws inspiration from the morphological structure of the diving beetle’s hindfoot, including the femur, tibia, tarsus, swimming hairs, and corresponding joints and limiting grooves. The swimming hairs in the hindfoot structure of diving beetles can be designed as flexible passive swimming fins, while the joints can be designed as flexible passive joints. This structure can provide stable support and generate significant propulsion, enabling the robot to perform movements similar to those of diving beetles during swimming, thereby allowing the robot to move efficiently through water.

**Figure 4:**
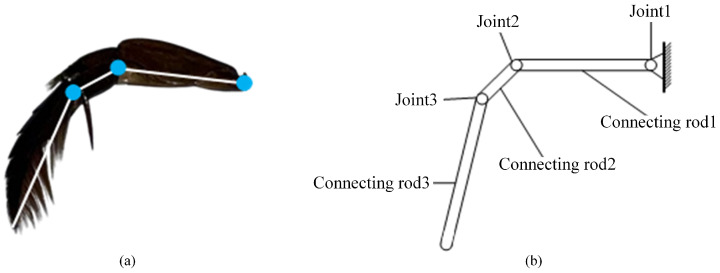
Schematic diagram of swimming foot structure: (**a**) Hindfoot construction. (**b**) Sketch of the structure of a robotic swimming foot.

The diving beetle’s swimming foot can be simplified into a structure comprising three connecting rods and three joints. As illustrated in Figure 4, the trochanter, where it connects to the diving beetle’s body, is designated as Joint 1 and is designed as an active joint to facilitate the swinging motion of the hindfoot. The segment between the trochanter and the femur is defined as connecting rod 1. The connection between the femur and the tibia is labeled as Joint 2, and the segment between the tibia and the tarsus is designated as connecting rod 2. Joint 3 is located between the tibia and the tarsus, with the entire tarsus serving as connecting rod 3. Both Joint 2 and Joint 3 are designed as passive joints, ensuring smooth and efficient movement of the simplified structure. This anatomical breakdown and functional assignment provide a clear and concise understanding of the diving beetle’s swimming foot structure and its mechanical implications.

**Figure 5:**
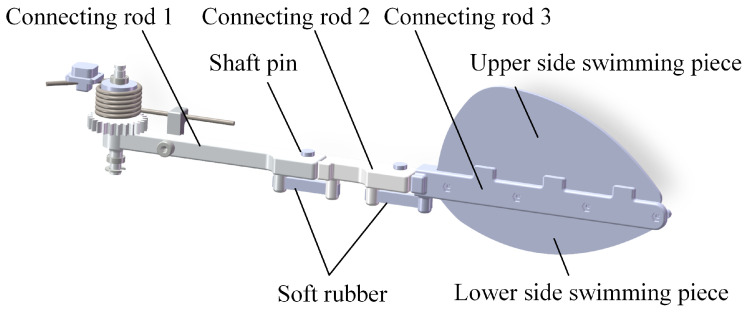
Imitation of diving beetle swimming feet decomposition diagram.

Based on the deployment characteristics of the diving beetle’s swimming hairs during swimming, the outer contour curve of the swimming hairs when they are deployed was captured. After smoothing the curve, the corresponding swimming fin was designed in proportion. Inspired by the hindfoot motion characteristics of the diving beetle, a swimming foot mechanism was designed to imitate the diving beetle’s swimming foot, as shown in Figure 5. This mechanism consists of several key components: connecting rod 1, imitating the diving beetle’s femur; connecting rod 2, imitating the tibia; connecting rod 3, imitating the tarsus; and the swimming fin, imitating the diving beetle’s swimming hairs. The length ratios of connecting rod 1, connecting rod 2, and connecting rod 3 are 9:4.5:13, consistent with the proportions of the diving beetle’s legs. Additionally, soft rubber is used to provide joint stiffness, along with connecting components such as pins, snap rings, and others.

The front end of connecting rod 1 is designed with a gear, whose primary function is to engage with the drive mechanism and facilitate power transmission. The rear end of connecting rod 1 is equipped with a groove that interfaces with the front end of connecting rod 2, and the two are securely connected using pins and retainers, enabling rotational movement between connecting rod 1 and connecting rod 2. The groove on the rear end of connecting rod 1 and the protrusion on the front end of connecting rod 2 are designed to restrict the swinging motion of the connecting rods, ensuring that they can only swing in a single direction. The same connection method is applied between connecting rod 2 and connecting rod 3. The swimming fin is secured to connecting rod 3 using bolts, and connecting rod 3 features three protrusions that serve to limit the position of the swimming fin.

**Figure 6:**
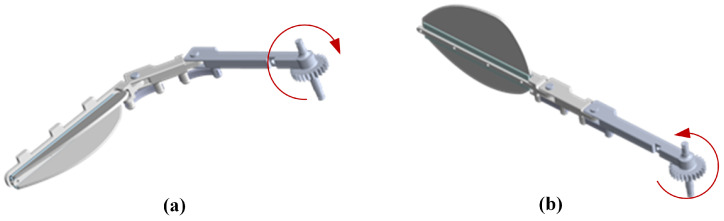
Schematic diagrams of the swimming process of the swimming foot: (**a**) The swimming foot return schematic. (**b**) The swimming foot stroke schematic.

Figure 6a illustrates the structural morphology of an imitation of the diving beetle swimming foot during the return phase: the swimming foot swings forward, and the soft rubber at the joints of the connecting rods, along with the bending of the swimming fins on both sides, reduces the overall contact area between the swimming foot and water, thereby effectively lowering the resistance acting on the swimming foot. As shown in Figure 6b, during the stroke phase, the swimming foot swings backward, and the joints of the connecting rods, as well as the swimming fins on both sides, rapidly extend. Due to the limiting structure, the swimming foot maintains its extended state, which increases the contact area with water and enhances the propulsive force during the swimming foot’s stroke. Through this design, the imitation of the diving beetle swimming foot can mimic the swimming characteristics of the diving beetle’s hindfoot, achieving efficient propulsion in water.

**Figure 7:**
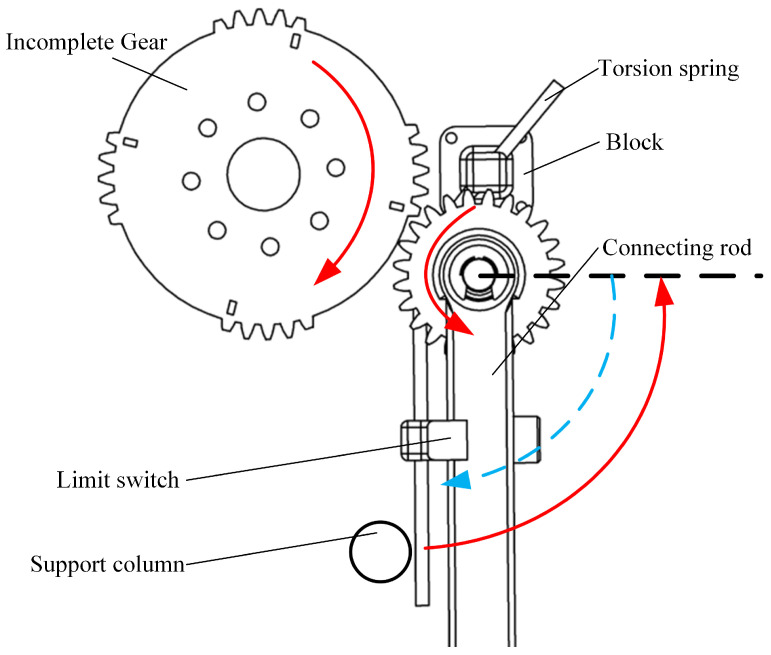
Transmission diagram of single-sided paddle propulsion mechanism.

Through the application of gear-driven transmission, the swimming propulsion mechanism can achieve higher swimming frequencies. However, during the two phases of movement, the diving beetle exhibits different stroke directions. If a full-tooth gear transmission were used, the servos would need to oscillate back and forth, with varying swing frequencies in different stages within a single cycle, resulting in a highly complex control system. To address this challenge, the present study employs an incomplete gear combined with a torsion spring for the transmission mechanism, as illustrated in Figure 7. This approach allows the servos to rotate in only one direction, thereby simplifying the control system. During gear engagement (red phase), the swimming foot completes the return stroke, while during the non-engagement phase, the torsion spring releases its pre-stored energy (blue phase), driving the swimming foot to complete the power stroke. This design enables the propulsion mechanism to achieve high-frequency swimming while reducing the required servo drive frequency, ultimately enhancing swimming efficiency.

**Figure 8:**
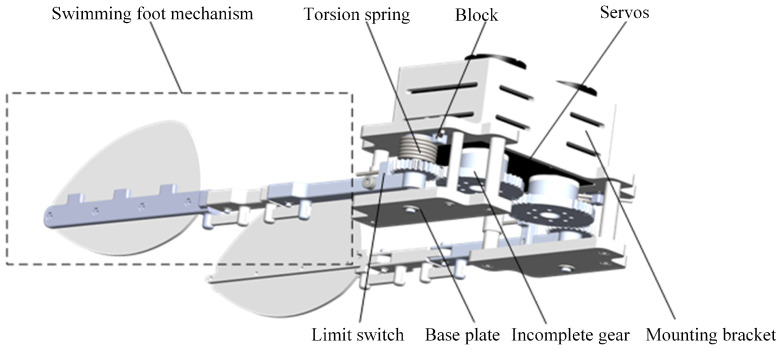
Paddle propulsion mechanism.

The designed components are integrated into the paddling propulsion mechanism, as shown in Figure 8. The paddling propulsion mechanism primarily consists of the swimming foot mechanism, torsion spring, incomplete gear, servos, mounting bracket, base plate, bearings, and other components. Among these, the mounting bracket serves to secure and support the various parts of the paddling propulsion mechanism. One end of the torsion spring is fixed in the block hole of the mounting bracket, while the other end is secured to connecting rod 1 via a limit switch. This design ensures the stability and robustness of the torsion spring while enabling it to provide the necessary torque for the paddling propulsion mechanism.

**Figure 9:**
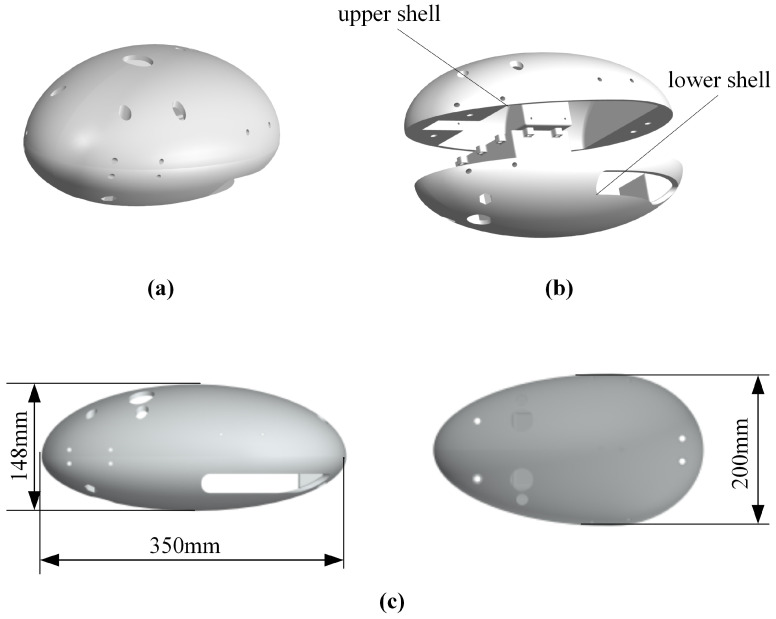
Shell design of paddle propulsion robot: (**a**) Schematic diagram of robot shell. (**b**) Robot shell decomposition schematic. (**c**) Dimensional drawings of housing.

**Figure 10:**
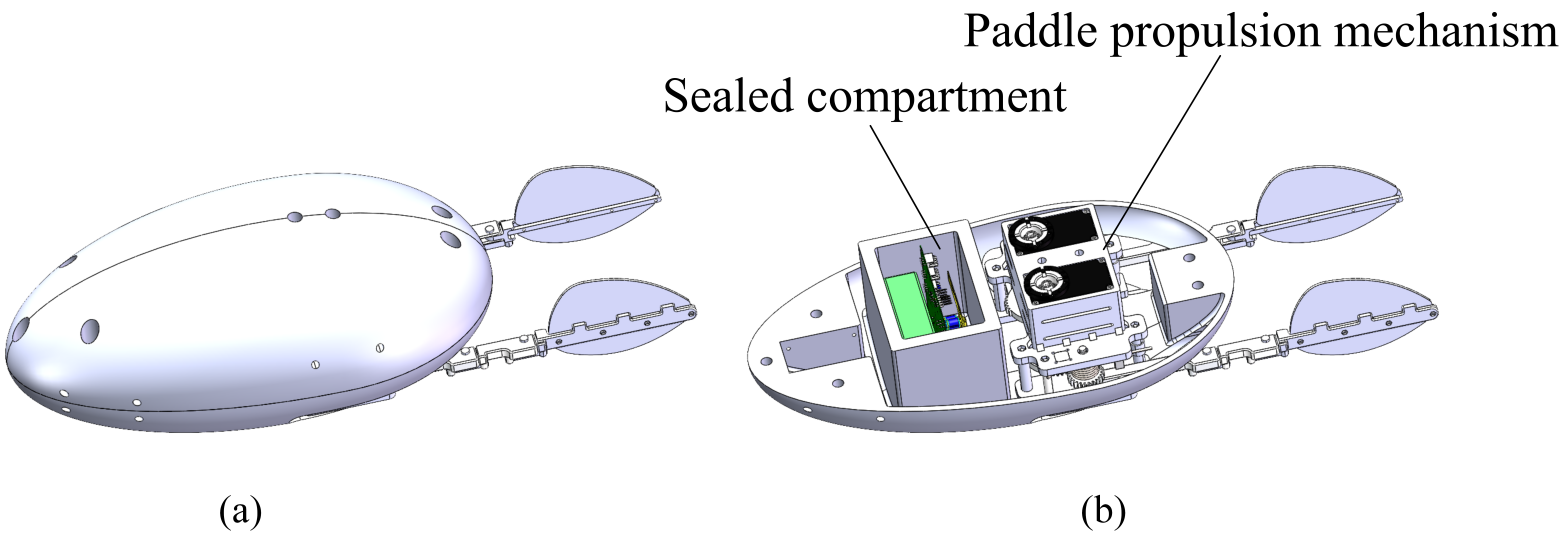
A three-dimensional diagram of the overall structure of the paddle propulsion robot: (**a**) The overall structure of the robot. (**b**) The internal structure of the robot.

Based on the diving beetle’s shape, a bio-inspired diving beetle robot shell was designed, as shown in Figure 9. The robot shell is composed of an upper and lower shell, with an overall streamlined shape to minimize fluid resistance. The upper shell is primarily used to secure the swimming propulsion mechanism, ensuring stability during robot movement and preventing swinging. The lower shell integrates with the waterproof enclosure, featuring a simple structure to reduce installation complexity. Figure 10 illustrates the overall structure of the bio-inspired diving beetle swimming robot. The sealed compartment houses the motion control board, batteries, and other electrical components, and the sealed compartment cover is bolted to the sealed compartment.

## 3. Dynamic Modeling

Deep investigation into the kinematics and wake behavior of diving beetles provides valuable insights for the design of bio-inspired devices [23,24]. In this section, the Lagrangian method is employed to establish a dynamic model for the unilateral paddling propulsion robot. Utilizing the Lagrangian approach for dynamic analysis enables the avoidance of computing interaction forces between individual components. Furthermore, with a generalized coordinate system as the reference, the relationship between forces and motion within the mechanism can be effectively elucidated. The Lagrangian dynamic model is expressed by Equation (1).

(1) $$\frac{d}{dt}\frac{\partial L}{\partial \dot{q}}-\frac{\partial L}{\partial q}=Q$$

where *L* represents the Lagrangian multiplier, defined as $L=E_{k}-E_{p}$; $q={\left[q_{1},q_{2},\ldots ,q_{n}\right]}^{T}$ represents the generalized coordinates; ${\left[{\dot{q}}_{1},{\dot{q}}_{2},\ldots ,{\dot{q}}_{n}\right]}^{T}$ represents the time derivatives of the generalized coordinates; $Q={\left[Q_{1},Q_{2},\ldots ,Q_{n}\right]}^{T}$ represents the generalized forces; and $E_{k}$ and $E_{p}$ denote the kinetic and potential energies of the mechanism, respectively.

### 3.1. Return Phase

**Figure 11:**
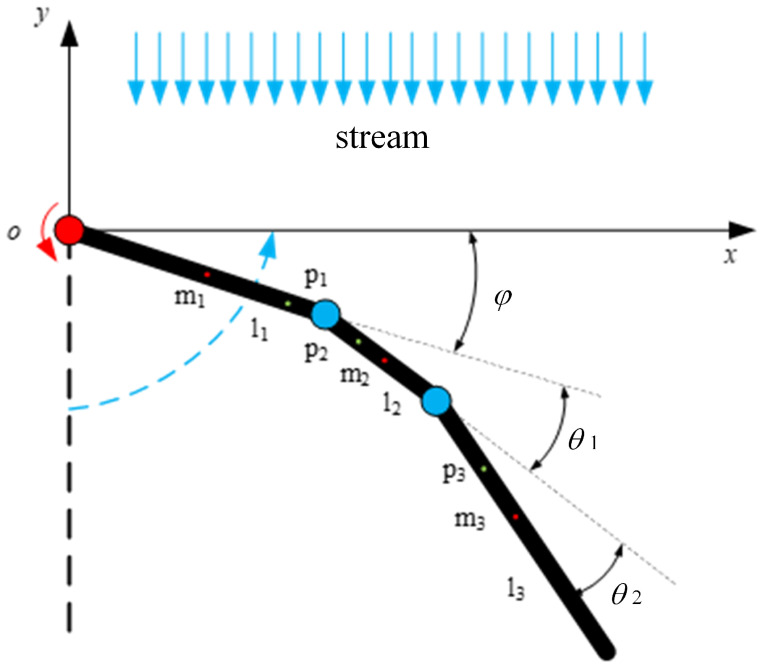
Dynamic model for return stage of unilateral paddle propulsion mechanism.

As shown in Figure 11, the front end of connecting rod 1 and point *O* at the active joint are taken as the coordinate origin, with O-xy serving as the reference coordinate system [25]. The servos drive the active joint to rotate by an angle φ, generating a torque $\tau_{\varphi }$. The rotation angles of the passive joints are denoted as $\theta_{i}$; the stiffness of the passive joints is denoted as $k_{i}$; the lengths of the connecting rods are denoted as $l_{j}$; the masses of the connecting rods are denoted as $m_{j}$; and the distance from the front end of each connecting rod to its center of mass is denoted as $l_{mj}$, where $l_{mj}=\frac{l_{j}}{2}$, with i=1,2 and j=1,2,3. The dynamic equation of the paddling propulsion mechanism is given by Equation (2):

(2) $$D\left(q\right)\ddot{q}+C\left(q,\dot{q}\right)\dot{q}=Q$$

Equation (2) is defined as follows: D(q)—symmetric positive definite inertia matrix; $C(q,\dot{q})$—a 3×3 matrix including centrifugal and Coriolis terms; and *Q*—generalized forces on institutions.

Equation (2) can be rewritten as shown in Equation (3):

(3) $$\left[\begin{array}{ccc} d_{11} & d_{12} & d_{13}\\ d_{21} & d_{22} & d_{23}\\ d_{31} & d_{32} & d_{33} \end{array}\right]\left[\begin{array}{c} \ddot{\varphi }\\ {\ddot{\theta }}_{1}\\ {\ddot{\theta }}_{2} \end{array}\right]+\left[\begin{array}{ccc} c_{11} & c_{12} & c_{13}\\ c_{21} & c_{22} & c_{23}\\ c_{31} & c_{32} & c_{33} \end{array}\right]\left[\begin{array}{c} \dot{\varphi }\\ {\dot{\theta }}_{1}\\ {\dot{\theta }}_{2} \end{array}\right]=\left[\begin{array}{c} \tau_{\varphi }\\ \tau_{1}\\ \tau_{2} \end{array}\right]$$

The corresponding coordinates of the center of mass of connecting rod 1 under the reference coordinate system are $\left(x_{m1},y_{m1}\right)$, the corresponding coordinates of the center of mass of connecting rod 2 are $\left(x_{m2},y_{m2}\right)$, and the corresponding coordinates of the center of mass of connecting rod 3 are $\left(x_{m3},y_{m3}\right)$. The joint rotational angles φ, $\theta_{1}$, and $\theta_{2}$ are set to be the generalized coordinates, respectively, of $q_{1}$, $q_{2}$, and $q_{3}$. Figure 11 shows the coordinates of each connecting rod center of mass in the reference coordinate system, as shown in Equation (4).

(4) $$\left\{\begin{array}{c} x_{m_{1}}=l_{m_{1}}\cdot \cos q_{1}\\ y_{m_{1}}=-l_{m_{1}}\cdot \sin q_{1}\\ x_{m_{2}}=l_{1}\cdot \cos q_{1}+l_{m_{2}}\cdot \cos\left(q_{1}+q_{2}\right)\\ y_{m_{2}}=-l_{1}\cdot \sin q_{1}-l_{m_{2}}\cdot \sin\left(q_{1}+q_{2}\right)\\ x_{m_{3}}=l_{1}\cdot \cos q_{1}+l_{2}\cdot \cos\left(q_{1}+q_{2}\right)+l_{m_{3}}\cdot \cos\left(q_{1}+q_{2}+q_{3}\right)\\ y_{m_{3}}=-l_{1}\cdot \sin q_{1}-l_{2}\cdot \sin\left(q_{1}+q_{2}\right)-l_{m_{3}}\cdot \sin\left(q_{1}+q_{2}+q_{3}\right) \end{array}\right.$$

The full differentiation of Equation (4) can be used to find the velocity of the center of mass of each connecting rod along the *x* and *y* directions, and the velocity components in each direction of the solution ${\dot{x}}_{m1},{\dot{y}}_{m1},{\dot{x}}_{m2},{\dot{y}}_{m2},{\dot{x}}_{m3},{\dot{y}}_{m3}$.

The model is a two-dimensional model, so only the kinetic energy of the mechanism is considered. The kinetic energy of each connecting rod of the paddling propulsion mechanism can be obtained from $E_{k}=\frac{mv^{2}}{2}+\frac{I\omega^{2}}{2}$, where *m* is the mass of each connecting rod; *v* is the combined velocity of the velocity components in each direction of each connecting rod, which can be obtained from $v^{2}={\dot{x}}_{m}^{2}+{\dot{y}}_{m}^{2}$; and *I* is the rotational inertia. The model has three connecting rods, and the rotational inertia of the *j*-th connecting rod about the axis through its center of mass can be expressed as $I_{j}=\frac{m_{j}l_{j}^{2}}{12}$, where j=1,2,3; ω is the angular velocity of each connecting rod. The kinetic energy of each connecting rod is $E_{k1}$, $E_{k2}$, and $E_{k3}$.

The Lagrange multiplier $L=E_{k1}+E_{k2}+E_{k3}$, according to the Lagrange formula, needs to be calculated for the derivatives of the generalized coordinates $q_{1}$, $q_{2}$, and $q_{3}$. The derivatives of the generalized coordinates $q_{1}$, $q_{2}$, and $q_{3}$ are then calculated for the time derivatives. The results of the above calculations are brought into Equation (1) to obtain the elements of the symmetric positive definite inertia matrices D(q) and $C(q,\dot{q})$ matrices in Equation (3). The elements in the $C(q,\dot{q})$ matrix can be obtained from the (first class) Christoffel symbols, which are shown in Equation (5):

(5) $$c_{ijk}=\frac{1}{2}\left(\frac{\partial d_{kj}}{\partial q_{i}}+\frac{\partial d_{ki}}{\partial q_{j}}-\frac{\partial d_{ij}}{\partial q_{k}}\right)$$

Combining the elements in the D(q) matrix, it is calculated using Equation (5). Each element $c_{11},c_{12},c_{13},c_{21},c_{22},c_{23},c_{31},c_{32},c_{33}$ in the matrix is determined accordingly.

During the oscillation process of the paddling propulsion mechanism, the forces and moments applied to each connecting rod will be different due to the differences in the velocity and direction of the water flow. In order to analyze and calculate this situation, the connecting rod is subdivided into countless small cells using the theory of foliation, and the motion state of the water flow within each cell is approximated as constant. By analyzing and calculating the water motion state in each cell, the force and moment generated on each cell are integrated and calculated, allowing the variation in the force and moment generated by the connecting rod to be derived. If we set any point on each connecting rod as $p_{1},p_{2},p_{3}$, then any point on each connecting rod in the reference coordinate system can be expressed as

(6) $$\left\{\begin{array}{c} p_{1}\left(\varepsilon \right)=\left[\begin{array}{c} a\cos q_{1}\\ -a\sin q_{1} \end{array}\right]\\ p_{2}\left(\varepsilon \right)=\left[\begin{array}{c} l_{1}\cos q_{1}+b\cos\left(q_{1}+q_{2}\right)\\ -l_{1}\sin q_{1}-b\sin\left(q_{1}+q_{2}\right) \end{array}\right]\\ p_{3}\left(\varepsilon \right)=\left[\begin{array}{c} l_{1}\cos q_{1}+l_{2}\cos\left(q_{1}+q_{2}\right)+c\cos\left(q_{1}+q_{2}+q_{3}\right)\\ -l_{1}\sin q_{1}-l_{2}\sin\left(q_{1}+q_{2}\right)-c\sin\left(q_{1}+q_{2}+q_{3}\right) \end{array}\right] \end{array}\right.$$

where a,b,c denote the lengths, which range from $\left(0,l_{1}\right]$, $\left(0,l_{2}\right]$, $\left(0,l_{3}\right]$. The velocity at any point on each connecting rod can be expressed as

(7) $$\left\{\begin{array}{cc} & {\dot{p}}_{1}\left(\varepsilon \right)=\left[\begin{array}{c} -a\sin q_{1}\cdot {\dot{q}}_{1}\\ -a\cos q_{1}\cdot {\dot{q}}_{1} \end{array}\right]\\ & {\dot{p}}_{2}\left(\varepsilon \right)=\left[\begin{array}{c} -l_{1}\sin q_{1}\cdot {\dot{q}}_{1}-b\sin\left(q_{1}+q_{2}\right)\cdot \left({\dot{q}}_{1}+{\dot{q}}_{2}\right)\\ -l_{1}\cos q_{1}\cdot {\dot{q}}_{1}-b\cos\left(q_{1}+q_{2}\right)\cdot \left({\dot{q}}_{1}+{\dot{q}}_{2}\right) \end{array}\right]\\ & {\dot{p}}_{3}\left(\varepsilon \right)=\left[\begin{array}{c} (-l_{1}\sin q_{1}\cdot {\dot{q}}_{1}-l_{2}\sin\left(q_{1}+q_{2}\right)\cdot \left({\dot{q}}_{1}+{\dot{q}}_{2}\right)-\\ c\sin\left(q_{1}+q_{2}+q_{3}\right)\cdot \left({\dot{q}}_{1}+{\dot{q}}_{2}+{\dot{q}}_{3}\right))\\ (-l_{1}\cos q_{1}\cdot {\dot{q}}_{1}-l_{2}\cos\left(q_{1}+q_{2}\right)\cdot \left({\dot{q}}_{1}+{\dot{q}}_{2}\right)-\\ c\cos\left(q_{1}+q_{2}+q_{3}\right)\cdot \left({\dot{q}}_{1}+{\dot{q}}_{2}+{\dot{q}}_{3}\right)) \end{array}\right] \end{array}\right.$$

The unit vector perpendicular to each connecting rod can be expressed as

(8) $$v_{1}=\left[\begin{array}{c} \sin q_{1}\\ \cos q_{1} \end{array}\right],v_{2}=\left[\begin{array}{c} \sin(q_{1}+q_{2})\\ \cos(q_{1}+q_{2}) \end{array}\right],v_{3}=\left[\begin{array}{c} \sin(q_{1}+q_{2}+q_{3})\\ \cos(q_{1}+q_{2}+q_{3}) \end{array}\right]$$

Then, the velocity perpendicular to the connecting rod can be given by ${\dot{p}}_{{⊥}_{j}}\left(\varepsilon \right)=\left({\dot{p}}_{j}\left(\varepsilon \right)\cdot v_{j}\right)v_{j}$, where j=1,2,3. Then, the hydrodynamic force [26,27] on any point on the connecting rod is

(9) $$f_{j}\left(\varepsilon \right)=-C_{n}\rho \left|{\dot{p}}_{{⊥}_{j}}\left(\varepsilon \right)\right|{\dot{p}}_{{⊥}_{j}}\left(\varepsilon \right)$$

where $C_{n}$ is the drag coefficient and ρ is the density of the fluid. The position vector of any cell on the connecting rod with respect to the joint can be expressed as

(10) $$r_{\varepsilon }^{j}\left(\varepsilon \right)=p_{j}\left(\varepsilon \right)-p_{\left(j-i\right)}\left(l_{\left(j-i\right)}\right)$$

where $p_{\left(j-i\right)}\left(l_{\left(j-i\right)}\right)$ denotes the position vector at the end of the j-i-th connecting rod, i.e., at the joint. Then, the hydrodynamic torque acting on the joint by any unit on the *j*-th connecting rod is

(11) $$M_{j}\left(\varepsilon \right)=r_{\varepsilon }^{j}\times f_{j}\left(\varepsilon \right)$$

Then, the torque applied to the joint can be expressed as

(12) $$\tau_{i}=-k_{i}\theta_{i}+\sum_{j=j-1}^{3}\int_{0}^{l_{j}}M_{j}\left(\varepsilon \right)d\left(\varepsilon \right)$$

### 3.2. Stroke Phase

**Figure 12:**
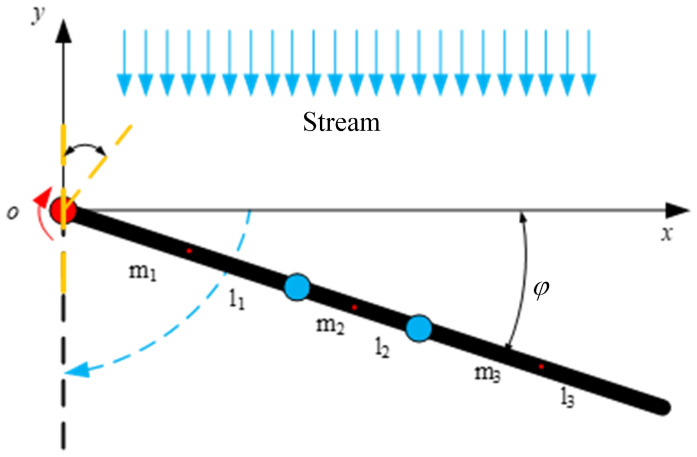
Dynamic model of stroke stage of unilateral paddle propulsion mechanism.

The paddling propulsion mechanism is designed as a finite structure so that the passive joints do not bend in the stroke phase, and thus the corresponding stiffness $k_{i}$ is assumed to be infinite when $\theta_{i}=0$. In the stroke stage, the paddling propulsion mechanism is modeled as a two-dimensional connecting rod with a rotary joint, and the connecting rod is driven to oscillate by the torsion spring, as shown in Figure 12. The length of the connecting rod is $l_{c}=l_{1}+l_{2}+l_{3}$, and its mass is $m_{c}$. Assuming that the mass of each connecting rod is concentrated at its midpoint, the distance from the leading end of the connecting rod to its center of mass is represented as $l_{mc}=\frac{l_{c}}{2}$. The free angle of the torsion spring is 180°, and the initial mounting angle is $\alpha =40^{\circ }$.

According to the Lagrange formula, its Lagrange multiplier can be expressed as

(13) $$L=E_{k}-E_{p}=\left(\frac{1}{2}m_{c}l_{m_{c}}^{2}+\frac{1}{24}m_{c}l_{c}^{2}\right)\cdot {\dot{q}}_{1}^{2}+\frac{1}{2}k_{s}{\left(\frac{13}{18}\pi +q_{1}\right)}^{2}$$

where $k_{s}$ is denoted as the stiffness of the torsion spring. Derivative calculations are performed for the generalized coordinates $q_{1}$, and the results of the derivatives of the generalized coordinates $q_{1}$, followed by the time derivatives, are expressed as follows:

(14) $$\left\{\begin{array}{c} \frac{\partial L}{\partial q_{1}}=k_{s}\left(\frac{13}{18}\pi +q_{1}\right)\cdot {\dot{q}}_{1}\\ \frac{d}{dt}\frac{\partial L}{\partial {\dot{q}}_{1}}=\left(m_{c}l_{m_{c}}^{2}+\frac{1}{12}m_{c}l_{c}^{2}\right)\cdot {\ddot{q}}_{1} \end{array}\right.$$

Setting any point on each connecting rod as $p_{c}$, the coordinates, velocity, and unit vector perpendicular to the connecting rod at any point on each connecting rod in the reference coordinate system can be expressed as

(15) $$\left\{\begin{array}{c} p_{c}\left(\varepsilon \right)=\left[\begin{array}{c} d\cos q_{1}\\ -d\sin q_{1} \end{array}\right]\\ {\dot{p}}_{c}\left(\varepsilon \right)=\left[\begin{array}{c} -d\sin q_{1}\cdot {\dot{q}}_{1}\\ -d\cos q_{1}\cdot {\dot{q}}_{1} \end{array}\right]\\ v_{c}=\left[\begin{array}{c} \sin q_{1}\\ \cos q_{1} \end{array}\right] \end{array}\right.$$

where *d* denotes the length, which ranges from $\left(0,l_{c}\right]$. Then, the velocity perpendicular to the connecting rod can be calculated by ${\dot{p}}_{{⊥}_{c}}\left(\varepsilon \right)=\left({\dot{p}}_{c}\left(\varepsilon \right)\cdot v_{c}\right)v_{c}$. The hydrodynamic force on any point on the connecting rod is

(16) $$f_{c}\left(\varepsilon \right)=-C_{n}\rho \left|{\dot{p}}_{{⊥}_{c}}\left(\varepsilon \right)\right|{\dot{p}}_{{⊥}_{c}}\left(\varepsilon \right)$$

The position vector $r_{c}=p_{c}\left(\varepsilon \right)$ of any cell on the connecting rod with respect to the position at the joint, and then the torque acting on the joint by the hydrodynamic force on any cell on the connecting rod is $M_{c}\left(\varepsilon \right)=r_{c}\times f_{c}\left(\varepsilon \right)$. According to the foliation theory, the torque applied to the joint can be expressed as

(17) $$\tau_{c}=\int_{0}^{l_{c}}M_{c}\left(\varepsilon \right)d\left(\varepsilon \right)$$

In summary, the kinetic equations of the unilateral paddling propulsion mechanism in the stroke phase can be expressed as

(18) $$\left(m_{c}l_{m_{c}}^{2}+\frac{1}{12}m_{c}l_{c}^{2}\right)\cdot {\ddot{q}}_{1}-k_{s}\left(\frac{13}{18}\pi +q_{1}\right)\cdot {\dot{q}}_{1}=\int_{0}^{l_{c}}M_{c}\left(\varepsilon \right)d\left(\varepsilon \right)$$

### 3.3. Simulation Data Analysis

We established a dynamic simulation model and conducted simulations by setting the servo driving frequencies to 0.1 Hz, 0.15 Hz, 0.2 Hz, 0.25 Hz, 0.3 Hz, and 0.35 Hz. Through the transmission system, the corresponding actual swimming foot oscillation frequencies were 0.4 Hz, 0.6 Hz, 0.8 Hz, 1 Hz, 1.2 Hz, and 1.4 Hz. In the figures of this paper, the frequencies are labeled using the servo driving frequencies, while the corresponding image frequencies represent the swimming foot oscillation frequencies. For example, when f = 0.25 Hz, the swimming foot oscillation frequency is 1 Hz, meaning one complete cycle is completed within 1 s. Therefore, in the figures, there should be 5 cycles within 5 s.

**Figure 13:**
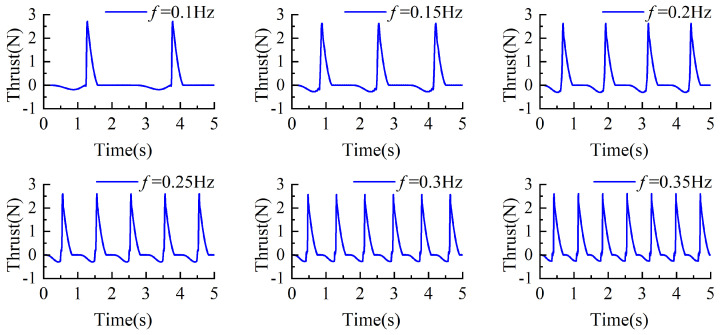
The change of thrust value under the same stiffness and different driving frequency.

Under the same torsion spring stiffness, the y-axis directional thrust corresponding to different driving frequencies is shown in Figure 13. Positive and negative values represent the direction of the thrust, where positive values indicate propulsive forces acting on the propulsion mechanism, and negative values indicate resistive forces. As depicted in the figure, during the returning phase, the swimming foot experiences resistance, and the magnitude of this resistance gradually increases. In contrast, during the stroke phase, the swimming foot generates thrust, which instantaneously increases and then diminishes as the swimming foot swings backward through a larger angle, until it returns to its initial position. Based on the data presented, higher driving frequencies result in greater resistive forces during the returning phase, while the effective propulsive forces generated during the stroke phase remain largely consistent.

**Figure 14:**
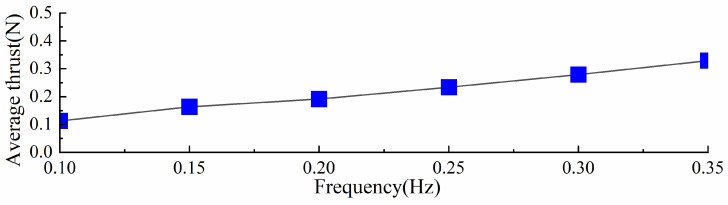
Different driving frequencies of average thrust with the same stiffness.

Under the same torsion spring stiffness, the stroke phase of the swimming foot exhibits similar hydrodynamic time spans across different driving frequencies. However, at lower frequencies, the time required for the swimming foot to return to its initial position and prepare for the next cycle is longer. Since the torsion spring stiffness remains constant, the maximum thrust generated during the stroke phase is approximately the same. Based on the thrust values obtained at different frequencies, the average thrust per cycle, referred to as average thrust, was calculated and is shown in Figure 14. It can be observed that as the driving frequency increases, the average thrust consistently increases. Therefore, under the same stiffness, increasing the driving frequency enhances the average thrust generated by the propulsion mechanism.

**Figure 15:**
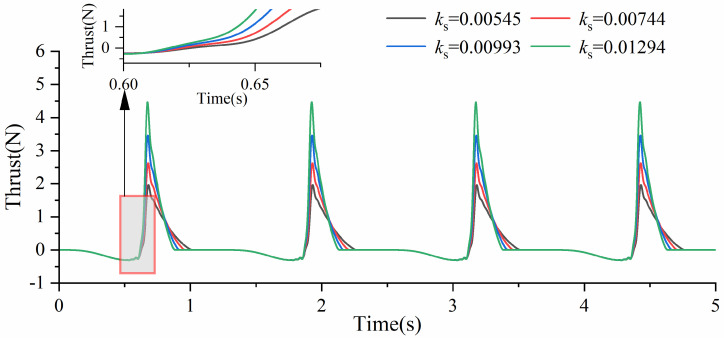
The change in thrust value of different stiffnesses.

As shown in Figure 15, under different torsion spring stiffness conditions and a driving frequency of 0.2 Hz, the thrust variation in the y-axis direction is presented for the swimming foot. During the returning phase, the swimming foot experiences consistent resistive forces. However, during the stroke phase, the thrust value increases more rapidly, and the maximum thrust value becomes larger as the torsion spring stiffness increases. This is because a higher torsion spring stiffness allows the swimming foot to swing backward faster, thereby accelerating the growth of thrust. Additionally, as the torsion spring stiffness increases, the time required for the swimming foot to complete its oscillation during the stroke phase decreases. From previous analyses, it is evident that when the time required for the returning phase is comparable to the actual oscillation time during the stroke phase, the swimming foot generates a higher average thrust. Therefore, increasing the torsion spring stiffness not only enhances the swimming foot’s thrust generation but also improves its propulsive efficiency. Furthermore, combining an increase in torsion spring stiffness with a higher driving frequency can effectively enhance the swimming foot’s propulsive performance, as it allows for a greater average thrust to be achieved.

**Figure 16:**
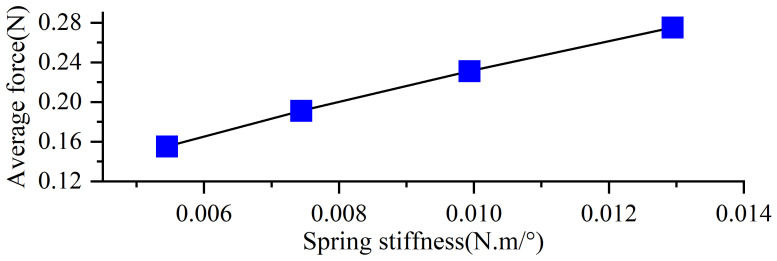
Average thrust of different stiffnesses.

As shown in Figure 16, the variation in average thrust generated by the swimming foot under different torsion spring stiffness conditions is presented. The results reveal that the average thrust consistently increases with the increase in the stiffness of the torsion spring [28]. Under the same driving frequency, increasing the spring stiffness effectively enhances the average thrust produced by the propulsion mechanism.

The simulation results demonstrate that both the driving frequency and spring stiffness significantly influence the thrust performance. Under the same spring stiffness, increasing the driving frequency can further elevate the average thrust. Additionally, with the same driving frequency, a higher torsion spring stiffness results in a faster increase in thrust values and achieves a larger maximum thrust.

## 4. Experiments and Results

The thrust testing and experimental research of the diving-beetle-inspired paddling propulsion robot can directly reflect the propulsion performance of the mechanism and the swimming performance of the robot [29]. This chapter establishes a thrust testing platform [30] and a swimming experiment platform, fabricates a single-sided paddling propulsion mechanism and a diving beetle-inspired robot, and conducts paddling thrust tests to measure the robot’s swimming performance under forward and turning gaits. The single-sided paddling propulsion mechanism was tested in a water tank with dimensions of 2000 mm × 1050 mm × 610 mm. The propulsion and swimming experiments were conducted in a larger water tank measuring 5.5 m × 1.8 m × 2 m.

### 4.1. Trials and Analysis of Unilateral Paddling Propulsion Mechanism Thrust Test

**Figure 17:**
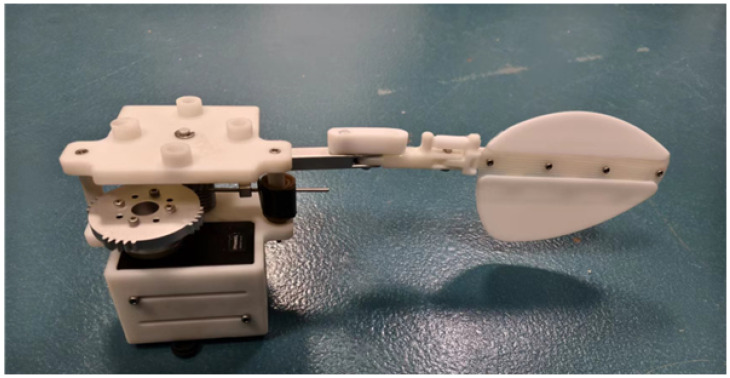
Prototype of single-sided paddle propulsion mechanism.

Based on the transmission system designed in this study, a torsion spring with a stiffness of 0.00744 Nm/° was employed to investigate the effects of different driving frequencies on the average thrust generated by the single-sided paddling propulsion mechanism under the same stiffness condition. The driving frequency used in the experiments was consistent with that in the simulations. The propulsion mechanism was installed on a force sensor (APDW-6D-110 EPIC, Anhui, China) fixed in a water tank, and the data collected by the sensor were analyzed to evaluate the average thrust performance (Figure 17).

**Figure 18:**
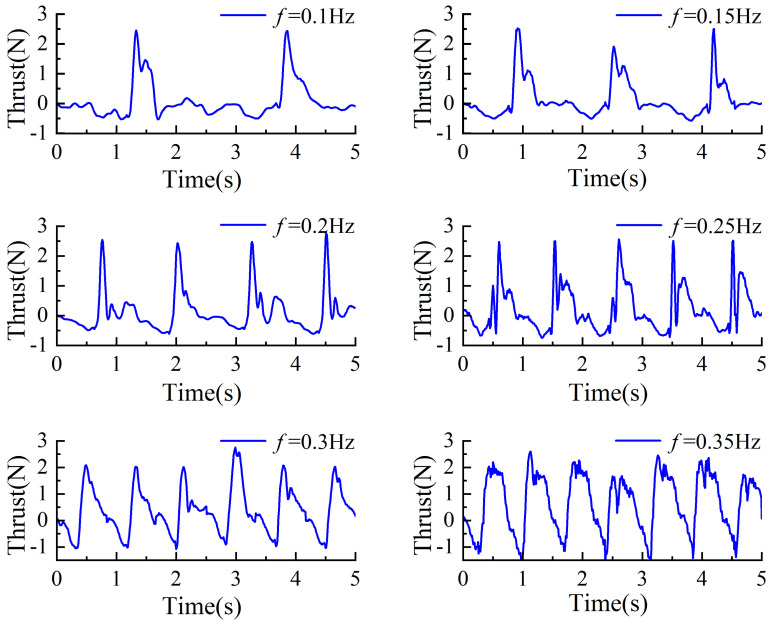
The change in thrust value of the same stiffness but different driving frequencies.

During the experimental process, the driving frequencies of the servos were set to be consistent with those used in the simulations, specifically configured at 0.1 Hz, 0.15 Hz, 0.2 Hz, 0.25 Hz, 0.3 Hz, and 0.35 Hz. Under these settings, the corresponding actual oscillation frequencies of the swimming foot were measured as 0.4 Hz, 0.6 Hz, 0.8 Hz, 1.0 Hz, 1.2 Hz, and 1.4 Hz, respectively. The average thrust generated in the forward direction was measured under each of these frequency conditions. The experimental results are illustrated in Figure 18, demonstrating the relationship between driving frequency and average thrust performance.

Based on the experimental results, the thrust variation rule of the propulsion mechanism during different phases can be summarized as follows: As the driving frequency increases, the resistance encountered by the propulsion mechanism during the return phase gradually increases. It is worth noting that the thrust generated during the stroke phase is primarily provided by the torsion spring. Therefore, under the same torsion spring stiffness, the actual stroke time of the swimming foot during the stroke phase remains constant, i.e., the time required for the swimming foot to move from its initial position to the rear position and back to the initial position is identical. Consequently, within this constant time frame, the magnitude of the generated thrust remains largely consistent. However, as the driving frequency decreases, the proportion of actual stroke time within the entire stroke phase diminishes. Therefore, at lower driving frequencies, the average thrust generated during the stroke phase decreases accordingly.

**Figure 19:**
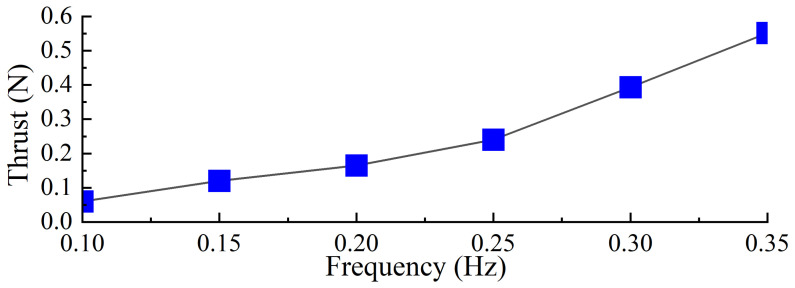
The average thrust value in one cycle for the same stiffness with different drive frequencies.

Based on the experimental results, the average thrust generated by the single-sided propulsion mechanism during a single cycle was calculated for different frequencies, as shown in Figure 19. The results indicate that as the driving frequency increases, the average thrust produced by the propulsion mechanism also increases. The experimental phenomena and the trend of thrust variation are consistent with the previously simulated trends. Combining Figure 18, it can be observed that the actual stroke time of the swimming foot during the stroke phase remains constant. However, as the driving frequency increases, the proportion of effective stroke time within the stroke phase increases. Although the resistance encountered by the swimming foot during the return phase increases with frequency, the magnitude of this increase is relatively small compared to the influence on the stroke phase. Therefore, as the driving frequency increases, the average thrust generated by the propulsion mechanism increases accordingly.

**Figure 20:**
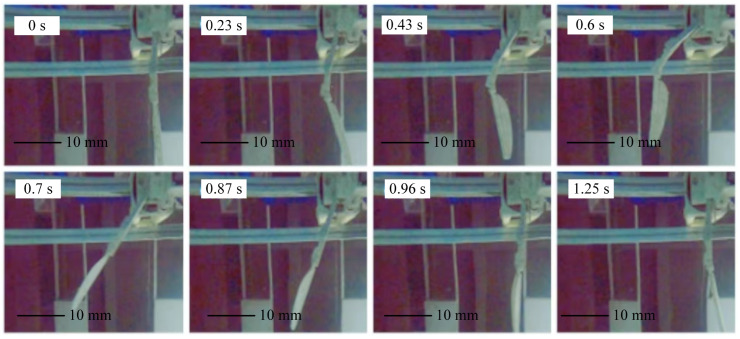
Motion state diagram of single-sided paddle propulsion mechanism.

The motion status of the swimming foot during water oscillation was recorded using a high-speed waterproof camera. As an example, at a driving frequency of 0.2 Hz, the motion status is shown in Figure 20. The swimming foot completes a full cycle in 1.25 s, with the return phase (0–0.63 s) lasting 0.63 s and the stroke phase (0.63–1.25 s) lasting 0.62 s. The actual water-paddling time (i.e., the effective paddling time of the swimming foot) is 0.36 s.

**Figure 21:**
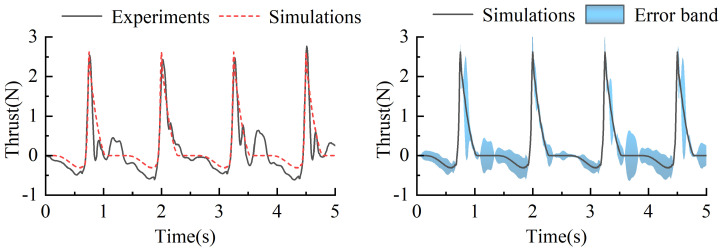
Change in thrust in test and simulation of single-sided paddle propulsion mechanism of 0.2 Hz.

At a frequency of 0.2 Hz, the thrust test results were compared with the simulation results, as shown in Figure 21. The thrust trends from both experiments and simulations exhibited similar variations. During the return phase, the swimming foot experienced resistance, while during the stroke phase, it generated thrust. Although discrepancies existed between the experimental and simulated thrust values, the differences were relatively small. Specifically, during the return phase, the simulated values were slightly smaller than the experimental ones, with an average error of 0.08 N. However, during the stroke phase, the thrust values were nearly identical. The primary reason for the minor discrepancies was the generation of water waves during the experimental paddling motion, which affected the force measurements and introduced slight deviations between the experimental and simulated thrust values. Despite these minor differences, the overall trends and numerical variations were consistent, verifying the accuracy and reliability of the simulation model.

### 4.2. Swimming Test and Analysis of Paddling Propulsion Robots

**Figure 22:**
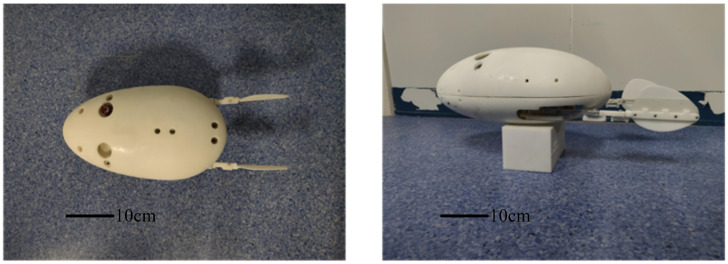
Prototype of diving beetle paddle propulsion robot.

To investigate the influence of different driving frequencies on the swimming performance of the biomimetic underwater robot (Figure 22) under the same torsional stiffness (0.00744 Nm/°), experiments were conducted with the robot performing various swimming gaits, including straight swimming and turning maneuvers. The robot’s swimming speed, turning speed, and turning radius were measured to evaluate its locomotive performance in water across different swimming modes.

#### 4.2.1. Forward Swimming Experiment

**Figure 23:**
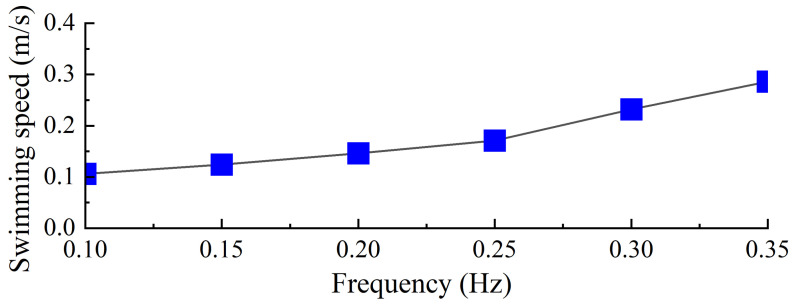
Forward swimming speed of robot.

**Figure 24:**
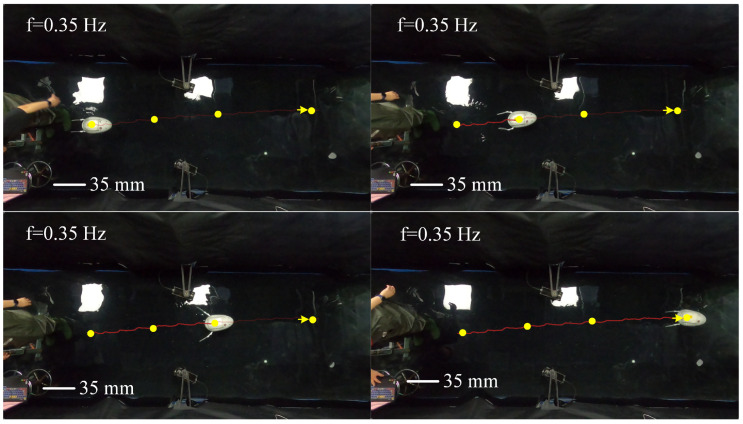
Process of forward swimming.

To evaluate the swimming performance of the biomimetic underwater robot under a torsional stiffness of 0.00744 Nm/°, experiments were conducted to investigate the effects of different driving frequencies on its propulsive swimming performance. The robot’s swimming speed was measured under various swimming gaits, including forward swimming and turning maneuvers. As illustrated in Figure 23, the swimming speed of the robot was recorded at different frequencies, with the forward swimming process at 0.35 Hz shown in Figure 24. Data processing and analysis were performed using motion analysis software to quantify the robot’s locomotive performance.

Among the tested driving frequencies, the robot achieved its maximum swimming velocity of 0.82 BL/s at a servo driving frequency of 0.35 Hz. As shown in Figure 18, an increase in driving frequency resulted in a higher average thrust generated by the swimming propulsion mechanism, and this trend was consistent with the variation in the robot’s swimming velocity. Considering the relatively large mass of the robot, the ratio of speed to mass is more meaningful. Our robot has a mass of 2.6 kg and a ratio of speed to mass of 0.315 BL/s per kg. The frog-inspired swimming robot has a swimming speed of 0.43 BL/s [13]. Compared to it, our diving-beetle-inspired robot swims faster.

**Figure 25:**
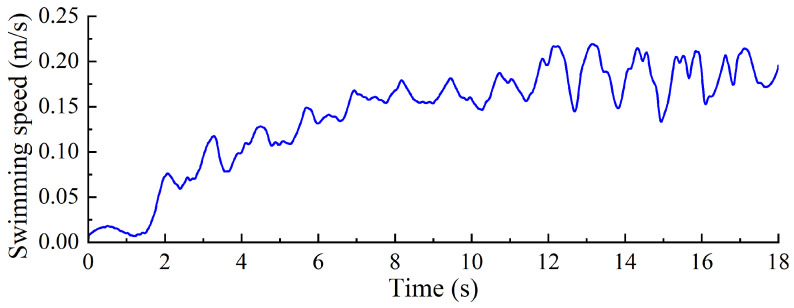
Change in swimming speed of diving beetle paddle propulsion robot.

As shown in Figure 25, the relationship between swimming velocity and time was measured for the swimming propulsion robot under a servo driving frequency of 0.2 Hz. The velocity begins at 0 m/s and gradually increases, reaching a relatively stable value of 0.175 m/s. Thereafter, the velocity fluctuates slightly around this value. The velocity curve exhibits a wavelike pattern, characterized by a periodic trend of increasing, decreasing, and then increasing again. This variation is primarily attributed to the two distinct motion phases in the swimming propulsion mechanism’s operating cycle: the “stroke” phase and the “return” phase. During the stroke phase, the propulsion mechanism generates thrust by moving backward, which accelerates the robot. Conversely, during the return phase, the mechanism moves forward, encountering water resistance, which decelerates the robot. Consequently, the robot’s velocity fluctuates accordingly. Throughout the swimming process, the robot’s velocity increases from 0 and stabilizes at a certain level after reaching a specific threshold. Compared to the frog-inspired swimming propulsion robot [13], the diving-beetle-inspired swimming propulsion robot demonstrates a relatively higher swimming velocity, as evidenced by the experimental results.

#### 4.2.2. Turning Swimming Experiment

**Figure 26:**
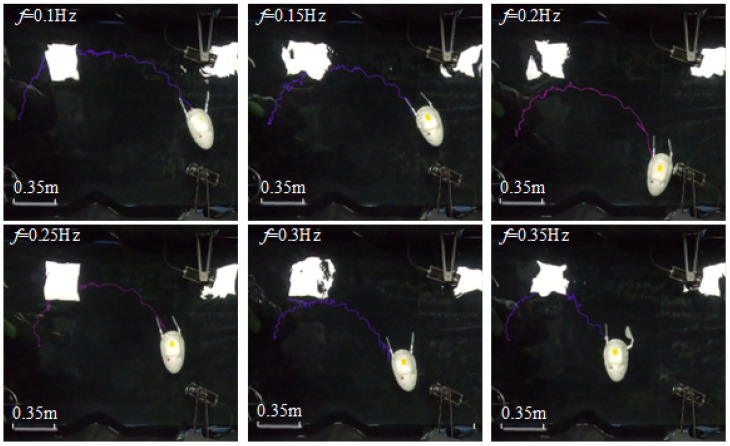
Walking path of robot turning gait with different driving frequencies.

This robotic design exhibits axial symmetry in its overall structure; consequently, the swimming performance metrics, including swimming velocity and turning speed, are similar for both left and right turning gaits. This investigation focuses on the right-turning gait as a representative case for analysis. Consistent with the previous experimental setup, the driving frequencies for the servos during the experiments were maintained. The swimming states of the robot under different frequencies during the right-turning gait are illustrated in Figure 26.

As shown in Figure 26, when rotating 180 degrees, the distance between the robot’s starting and ending points decreases as the driving frequency increases. At lower frequencies, the robot’s forward speed is slower, and the time required for turning is longer, resulting in a larger turning path. As the driving frequency increases, both the robot’s forward speed and turning efficiency improve, thereby reducing the turning path. The shortest turning distance (from the starting point to the ending point) is achieved at a driving frequency of 0.35 Hz, measuring 0.985 m.

**Figure 27:**
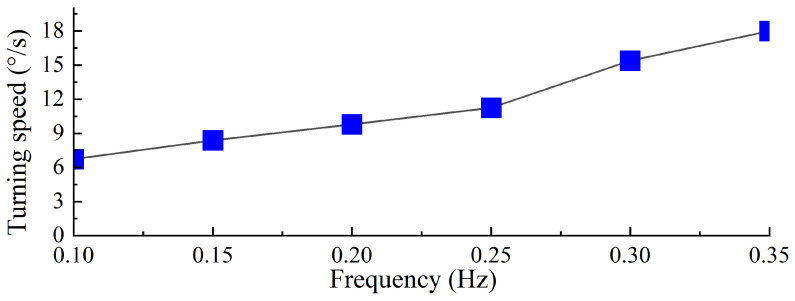
Turning speed of diving beetle paddle propulsion robot with different driving frequencies.

As shown in Figure 27, based on the swimming data of the right-turning gait at different driving frequencies, combined with video data and motion analysis software, the turning speed of the biomimetic diving beetle propulsion robot under different frequencies was calculated and measured. The results indicate that as the driving frequency increases, the turning speed of the robot also increases. At a driving frequency of 0.35 Hz, the robot achieves its maximum turning speed of 18°/s. The increase in driving frequency significantly enhances the robot’s turning capability, and within a certain range, the turning speed increases with the driving frequency. Compared to the frog-inspired propulsion robot [13], the biomimetic diving beetle propulsion robot demonstrates a faster turning speed.

## 5. Conclusions

This paper investigates the structural characteristics and swimming properties of the diving beetle’s swimming legs and, based on this, designs a biomimetic diving beetle swimming propulsion robot. The robot employs a transmission method combining an incomplete gear transmission and torsion spring, which reduces the driving frequency of the servos and simplifies the control of the swimming robot. Additionally, the robot achieves specific motion patterns during the return and stroke phases, enhancing its biomimetic degree and swimming performance. To describe its dynamic characteristics, this study establishes a two-dimensional, three-joint serial robotic arm dynamic model with passive properties by combining Lagrangian mechanics and blade element theory. This approach ensures the reliability of the model while reducing computational complexity. Through simulation experiments and underwater experiments, the swimming performance data of the biomimetic robot were collected and analyzed to explore the effects of different driving frequencies on its swimming propulsion capability. The results demonstrate that when the servo motor driving frequency is 0.35 Hz, the robot achieves its highest swimming speed, reaching 0.82 BL/s. This research verifies the swimming reliability of the biomimetic diving beetle swimming propulsion robot and provides significant references for the design and optimization of small-scale biomimetic swimming propulsion robots.

In future work, we can explore the use of more compact actuators, lightweight materials, and optimized designs to achieve size reduction without compromising performance. Additionally, bio-inspired solutions, such as flexible materials or alternative propulsion mechanisms, may be investigated to further enhance miniaturization possibilities. And we plan to explore the robot’s underwater maneuverability and diving capabilities in greater depth.

## Data Availability

The datasets generated during and/or analyzed during the current study are available from the corresponding author on reasonable request.
